# Nationwide assessment of early hospitalizations and mortality after pulsed field ablation

**DOI:** 10.1093/europace/euag300

**Published:** 2026-10-21

**Authors:** Pavel Osmancik, Petr Peichl, Stepan Havranek, Jan Petru, Jiri Hanis, Ludek Haman, Veronika Bulkova, Anna Rehakova, Jiri Jarkovsky

**Affiliations:** Department of Cardiology, 3rd Faculty of Medicine, University Hospital Kralovske Vinohrady, Charles University, Ruska 87, 10000 Prague, Czech Republic; Department of Cardiology, Institute for Clinical and Experimental Medicine, Prague, Czech Republic; 1st Clinic of Cardiology and Angiology, 1st Faculty of Medicine, General Teaching Hospital, Prague, Czech Republic; Department of Cardiology, Na Homolce Hospital, Prague, Czech Republic; Department of Cardiology, Regional Hospital Ceske Budejovice, Ceske Budejovice, Czech Republic; Department of Cardiology, University Hospital Hradec Kralove, Hradec Kralove, Czech Republic; Department of Cardiology, NEURON Medical Center, Brno, Czech Republic; Institute of Health Information and Statistics of the Czech Republic, Brno, Czech Republic; Institute of Health Information and Statistics of the Czech Republic, Brno, Czech Republic; Institute of Biostatistics and Analyses, Masaryk University, Brno, Czech Republic

**Keywords:** Atrial fibrillation, Catheter ablation, Pulsed field, Mortality, Complication

## Introduction

Catheter ablation (CA) for atrial fibrillation (AF) using pulsed field (PF) energy represents an emerging modality for AF ablation.^1–3^ Although data from large landmark registries have demonstrated low mortality associated with PF ablation (PFA), some recent reports have raised concerns about a risk of early mortality and sudden cardiac death (SCD) following PFA.^4–7^

In the Czech Republic, all CA procedures are entered into a mandatory nationwide registry, which since 2024 has been linked to both national mortality registry and hospitalization records from all healthcare providers. The aim of the study was to analyse early mortality and hospitalizations in patients following PFA for AF.

## Methods

The study was approved by the Ethics Committee of University Hospital Kralovske Vinohrady (VV/102/00/2026) and was conducted in accordance with Strengthening the Reporting of Observational Studies in Epidemiology guidelines. Initially, the mandatory nationwide database of the Institute of Health Information and Statistics of the Czech Republic was analysed to identify all patients who underwent PFA for AF in 2024 and to obtain baseline clinical characteristics. Through linkage with national health insurance registries and national mortality registry, data on re-hospitalizations, including admission diagnoses, and all-cause mortality within 28 days of the procedure were obtained. For re-hospitalizations, only the date of re-admissions and diagnosis codes were available. For deceased patients, the date, place of death, and diagnosis codes were available. The respective centres were identified and asked to provide fully anonymized clinical records, including details of the re-hospitalization course, the ablation, and cause of death. Continuous variables are presented as median and interquartile range and categorical variables as counts and/or percentages.

## Results

Between 1 January 2024 and 31 December 2024, a total of 6750 PFA procedures for AF were performed in 6624 patients. The median age was 66 (58–72) years; 35.4% of patients were female, and 48.0% had paroxysmal AF. Heart failure (HF), hypertension, and diabetes were present in 13.4%, 62.7%, and 18.1% of patients, respectively. The Farapulse system (Boston Scientific, USA) was used in 98.6% of procedures.

### 28-day morbidity

A total of 288 patients were re-hospitalized within 28 days following the procedure, corresponding to 4.35% of patients, or 4.27% of procedures. The temporal distribution of re-hospitalizations and deaths and the distribution of re-hospitalization diagnoses according to International Classification of Diseases codes are shown in *Figure 1A* and *B*. Cardiovascular conditions accounted for the majority of hospitalizations (61%). Sustained ventricular tachycardia (VT) was the primary reason for admission in only one patient.

**Figure 1:**
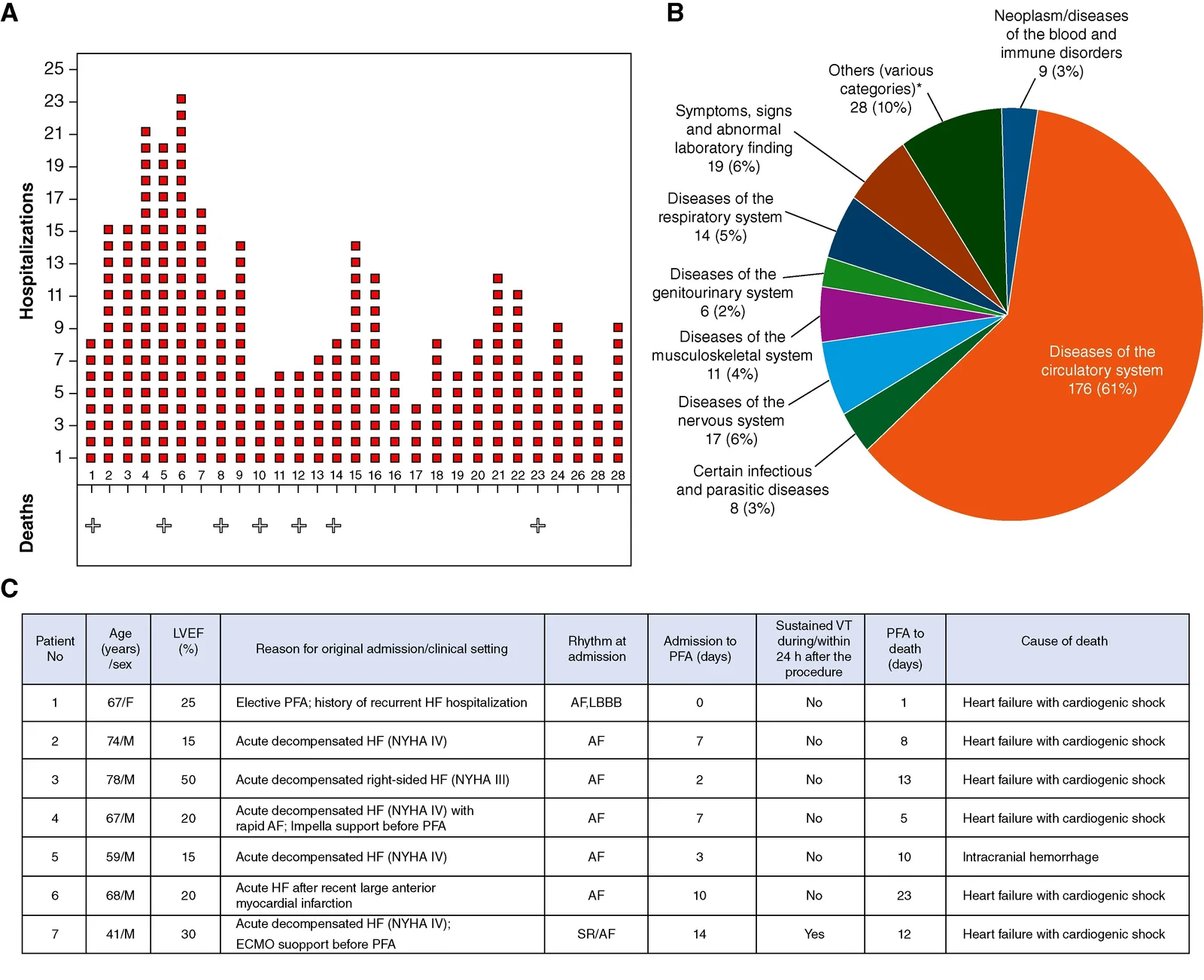
(*A*) Density plot showing the temporal distribution of re-hospitalizations (above the *x*-axis) and deaths (below the *x*-axis) during the 28-day follow-up period. (*B*) Pie chart showing admission diagnoses according to the International Classification of Diseases codes in patients hospitalized within 28 days of the procedure. (*C*) Clinical characteristics and course of patients who died within 28 days after PFA. F, female; M, male; LVEF, left ventricular ejection fraction; PFA, pulsed field ablation; AF, atrial fibrillation; LBBB, left bundle branch block; VT, sustained ventricular tachycardia; HF, heart failure; NYHA, New York Heart Association; SR, sinus rhythm; ECMO, extracorporeal membrane oxygenation.

### 28-day mortality

A total of seven patients died within 28 days of the procedure, corresponding to a mortality rate of 0.10% per patient and 0.11% per procedure. All seven procedures were performed using the pentaspline catheter. Death occurred a median of 10.0 days (5.0–13.0) after ablation. Causes of death were intracranial haemorrhage (*n* = 1) and progressive HF with cardiogenic shock (*n* = 6).

Detailed information regarding baseline characteristics, hospitalization, and clinical trajectories up to death is summarized in *Figure 1C*. Median age was 67.0 years (59.0–74.0), six were males, and left ventricular ejection fraction was 20.0% (15.0–30.0). Six patients suffered from persistent AF, whereas one patient had paroxysmal AF with recurrent AF episodes associated with haemodynamical deterioration. Six patients were admitted with acute decompensated HF before PFA and underwent ablation during the same hospitalization after haemodynamic stabilization. Among these patients, one had a recent myocardial infarction, one required Impella support, and one required ECMO support before ablation. The remaining patient underwent an elective ablation; however, he had a history of recurrent HF hospitalizations. Atrial fibrillation was considered contributory to HF deterioration in all seven patients. Heart failure aetiology was coronary artery disease (*n* = 1), dilated cardiomyopathy (*n* = 2), valvular heart disease (*n* = 1), hypertrophic cardiomyopathy (*n* = 1), and AF-induced cardiomyopathy (*n* = 2). Acute renal failure and sepsis as complications occurred in two patients each. Sustained VT requiring defibrillation occurred in one patient during the procedure; no other patient experienced sustained VT during the procedure, and no sustained VT was reported in any patient from the end of the procedure until death. None of the deaths was adjudicated as SCD.

## Discussion

The present study provides one of the first nationwide contemporary safety benchmarks for PFA in routine clinical practice. Early mortality following PFA for AF was very low (0.11%). All patients who died had advanced HF before ablation, most presenting with acute decompensated HF. No death was adjudicated as VT-related SCD or occurred in patients without structural heart disease.

Very low mortality following PFA has been reported in recent large-scale registries.^4,5^ However, unlike registries based on voluntary reporting by participating centres, our analysis is derived from a nationwide mandatory registry encompassing all ablation procedures performed in the Czech Republic in 2024, substantially reducing selection and reporting bias. Furthermore, linkage with national mortality and hospitalization databases enabled near-complete ascertainment of post-procedural clinical events.

A mortality benefit of CA in patients with advanced HF with reduced EF was demonstrated in the CASTLE-HTx trial.^8^ Importantly, all CASTLE-HTx patients were required clinically stable at randomization. It is in contrast to our cohort, in which most patients who subsequently died had been admitted with acute decompensated HF. Acute decompensated HF may therefore represent both a substantial competing risk for early mortality and a condition of increased vulnerability to procedural stress. Turagam *et al*. reported outcomes after PFA in 1381 AF patients, including 83 with HF with reduced EF, with no deaths reported within 7 days.^9^ It remains unclear whether ablations were elective or performed during acute HF admission, and only 7-day mortality was assessed. In our cohort, most patients were admitted with acute HF decompensation, and also importantly, the median time to death was 10 days. Therefore, our findings raise important questions regarding the clinical benefit–risk balance of performing PFA in decompensated HF patients.

Gunawardene *et al*. reported VT/SCD in 11 of 6721 patients (0.16%) undergoing PFA,^10^ mostly during or shortly after the procedure. In our cohort, sustained VT during the procedure occurred in only one patient. Collectively, these findings support the favourable safety profile of PFA, particularly in clinically stable patients.

## Data Availability

The data underlying this article were provided by the Institute of Health Information and Statistics of the Czech Republic. Data may be shared upon reasonable request to the corresponding author with permission of the Institute of Health Information and Statistics of the Czech Republic, provided that a clearly defined research protocol specifying the intended use of the data is submitted, the protocol has received approval from the relevant ethics committee, and the request has undergone appropriate legal review, given that the data originate from a nationwide patient database.
